# Network Pharmacology, Molecular Docking and Molecular Dynamics Studies to Predict the Molecular Targets and Mechanisms of Action of *Melissa officinalis* Phytoconstituents in Type-2 Diabetes Mellitus

**DOI:** 10.3390/plants14182828

**Published:** 2025-09-10

**Authors:** Chimaobi J. Ononamadu, Ziyad Ben Ahmed, Veronique Seidel

**Affiliations:** 1 Natural Products Research Laboratory, Strathclyde Institute of Pharmacy and Biomedical Sciences, University of Strathclyde, Glasgow G4 0RE, UK; ononamaducj@polac.edu.ng; 2 Natural Product Research Group, Department of Biochemistry and Forensic Science, Nigeria Police Academy, Wudil 713105, Kano State, Nigeria; 3 Laboratoire des Sciences Fondamentales, Université Amar Telidji, Laghouat 03000, Algeria; ziyad.ben.ahmed@vub.be; 4 Department of Analytical Chemistry, Applied Chemometrics and Molecular Modelling, Vrije Universiteit Brussel (VUB), 1050 Brussels, Belgium

**Keywords:** antidiabetic, *Melissa officinalis*, network pharmacology, molecular docking, type-2 diabetes mellitus

## Abstract

Network pharmacology, molecular docking, and molecular dynamics (MD) studies were used to investigate the molecular targets and mechanisms of action of *Melissa officinalis* phytoconstituents in type-2 diabetes mellitus (T2DM). SciFinder was used to retrieve previously known phytoconstituents from *M. officinalis* aerial parts. Targets related to these compounds were predicted using the Swiss TargetPrediction, SEA (similarity ensemble approach) and BindingDB databases, and were intersected with T2DM-relevant targets from public databases. Networks were constructed using the STRING online tool and Cytoscape (v.3.9.1) software. Gene ontology/KEGG pathway analysis was performed using DAVID and SHINEGO 0.77. Molecular docking used the MOE suite. MD simulations were conducted for 100 ns using GROMACS 2023 with a CHARMM36 force field. A total of 17 phytoconstituents and 154 targets associated with T2DM were identified. The protein–protein interaction (PPI) and target–pathway (TP) network analysis identified key hub genes, including EGFR, SRC, AKT1, TNF, PPARG, PIK3R1, RELA, INSR, GSK3B, PIK3CG, FYN, PTBIN, and PPARA, with critical roles in insulin resistance and T2DM-relevant pathways. The pathway enrichment analysis highlighted notable involvement in insulin signaling, inflammation, and diabetic complications. The compound–target (CT) network predicted quercetin, luteolin, ursolic acid, isoquercitrin, 2α-hydroxy-ursolic acid, and oleanolic acid to be key bioactive compounds. Molecular docking, followed by MD studies, identified that isoquercitrin showed most energetically favorable and stable complexes with three targets, namely EGFR, PPARα, and AKT1. These findings enhance our understanding of the antidiabetic potential of *M. officinalis* and underscore the need for further studies on its phytoconstituents, such as isoquercitrin, in search for new antidiabetic agents.

## 1. Introduction

Diabetes mellitus (DM) is a disorder of carbohydrate, protein, and lipid metabolism, primarily characterized by hyperglycemia and other metabolic anomalies. Its manifestation is usually discussed in the context of two major types, type-1 and type-2 diabetes mellitus (T2DM), with the latter being the most prevalent worldwide [1]. The chronic hyperglycemia in DM is associated with complications such as nephropathy, retinopathy, angiocardiopathy, cerebrovascular diseases, neuropathy, and even death [2,3]. From 2019 to 2021, the number of people living with DM rose from 463 million to 537 million [4,5]. These figures are predicted to further rise to 643 million and 783 million in 2030 and 2045, respectively. It has been reported that 75% of all DM cases occur in middle- and low-income countries, signifying an epidemic that demands immediate attention and interventions [5].

Whilst antidiabetic drugs have shown relative success in managing T2DM long-term, they are not without issues, including adverse side effects, toxicity, poor outcomes, and the inability to completely cure the condition [6]. Consequently, efforts to discover new antidiabetic agents continue to intensify, with many studies reporting on the investigation of plant medicines as sources of new drug templates [7,8,9,10]. The antidiabetic potential of several plants used for the management of DM has already been well-documented, especially in Africa, Asia, and parts of Europe [11,12].

*Melissa officinalis* L., commonly known as lemon or honey balm, is a perennial aromatic herb belonging to the Lamiaceae family [13]. It is commonly found in the Mediterranean region, Western Asia, and North Africa, and it is cultivated in many parts of Europe [14]. Lemon balm is a widely used medicinal and culinary plant [15]. It is employed in traditional medicine to treat various health conditions, such as mental, gastrointestinal, neurological, and rheumatoid disorders [16,17]. Different parts of the plant have been reported to improve cognitive ability [13] and have antidepressive, anxiolytic, sedative, antitumor, cytotoxic, antiproliferative, antimicrobial, and antioxidant effects [18,19,20,21,22,23]. In the Iranian Traditional Medicine system, *M. officinalis* is used for cardiovascular diseases, DM, and other associated metabolic disorders [24,25,26]. In vitro and in vivo studies have reported that lemon balm essential oil exhibited antinociceptive and antihyperglycemic activities, and was able to modulate lipid- and glycemic-regulating enzymes [15,24,27,28,29]. Various extracts from the aerial parts of *M. officinalis* have demonstrated hypoglycemic and hypolipidemic activities in animal models [26,27,28]. They have been reported to reduce glycation activity [29,30] and inhibit carbolytic enzymes [31]. Randomized clinical trials conducted in patients with T2DM and dyslipidemia have demonstrated that *M. officinalis* extracts could modulate glycemic flux, ameliorate hyperlipidemia [32], improve hyperglycemia, reduce cardiovascular risks [16,17], and reduce depression and anxiety [19]. Despite this body of evidence on the relevance of *M. officinalis* in T2DM, few studies have explored at once the multiple molecular targets and mechanisms of antidiabetic action of this plant.

The “one-compound–one target” model traditionally used to investigate the pharmacological mode of action of drugs/bioactive compounds at the molecular level is not suitable when dealing with diseases that have an intricate pathogenesis. This can be mainly explained by the fact that most complex diseases involve multiple targetable proteins/pathways that may be modulated by compounds/drugs with multimodal activities [1,33]. In this context, network pharmacology—which combines network biology and polypharmacology—has been recognized as a more appropriate approach for efficient and cost-effective drug discovery and development [34] since it offers a more integrated/holistic insight on the interactions between bioactive compounds and their various targets/pathways within a given biological system implicated in a disease condition [35,36]. Numerous studies have highlighted the successful application of network pharmacology and other computational techniques (e.g., molecular docking) in elucidating the multimodal activities of phytochemicals in complex disease conditions. These include cancer [37,38,39,40], DM [41,42,43,44] and its complications such as diabetic osteoporosis [45], and diabetic nephropathy [46,47]. One recent study explored the anticancer potential of *M. officinalis* using network pharmacology [48]. To the best of our knowledge, no work on the antidiabetic potential of *M. officinalis* using network pharmacology/molecular docking has ever been undertaken. The current study employed a network pharmacology and molecular docking-based approach to investigate the molecular targets and mechanisms of antidiabetic action of *M. officinalis* in T2DM.

## 2. Results

### 2.1. Compound Mining and Ranking

A total of 21 compounds were chosen based on a literature search for phytochemicals previously isolated from the aerial parts—leaves and stems—of *M. officinalis*. All compounds were deemed to meet the selection criteria and were thus considered for further analysis (Table S1).

### 2.2. Compound- and Disease-Associated Targets

**Figure 1:**
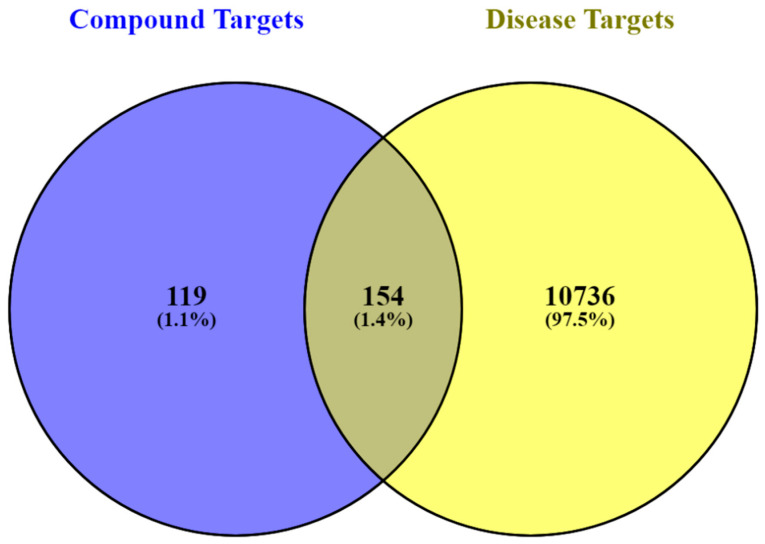
Venn diagram depiction of the biological targets common (intersection) to *M. officinalis* compounds and T2DM.

Among the 21 compounds, 17 were predicted to interact (probability score ≥ 0.60) with 275 biological targets following screening of the SEA, STP, and BindingDB databases, while a comprehensive search across the GeneCards, TTD, DisGeNET, KEGG, and Malacards databases yielded 10,890 targets associated with T2DM. A total of 154 targets were found to be related to both *M. officinalis* compounds and T2DM (Tables S2–S4; Figure 1).

### 2.3. Network Construction and Analyses

#### 2.3.1. Protein–Protein Interaction (PPI) Network

**Figure 2:**
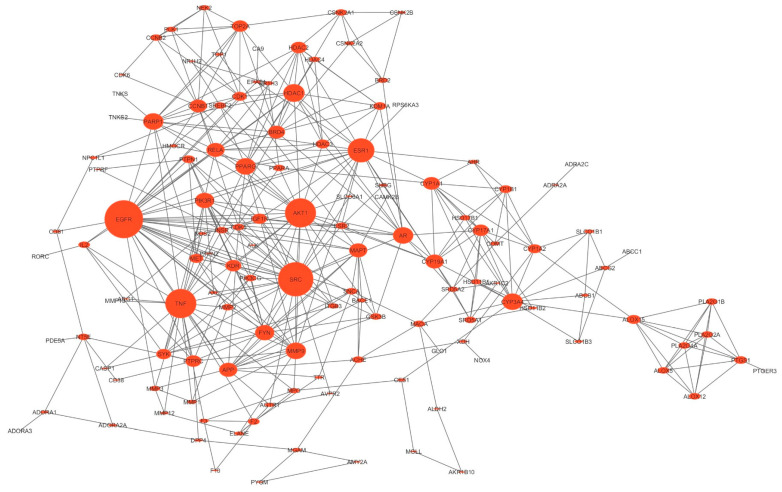
Protein–protein interaction (PPI) network with the sizes of the nodes proportional to the magnitude of the degree.

**Figure 3:**
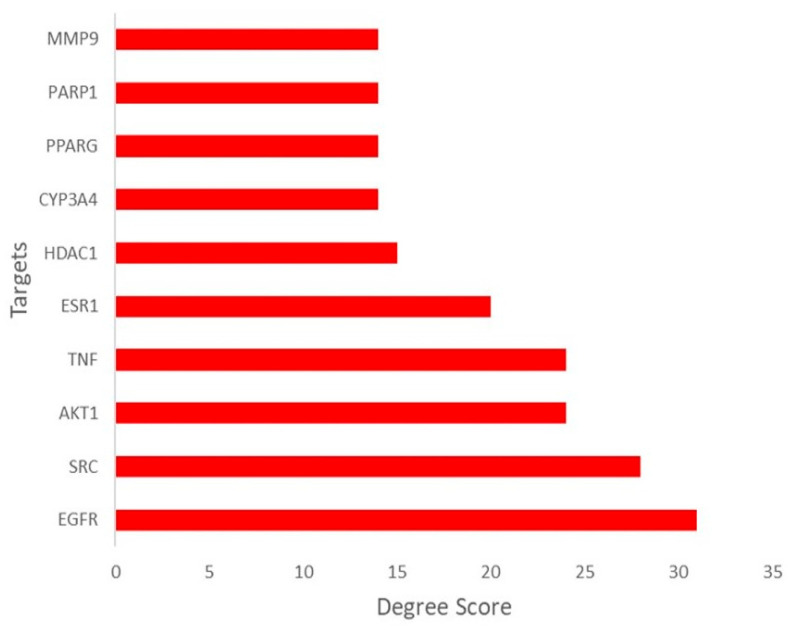
Top-10 ranked hub genes/T2DM targets of *M. officinalis* compounds.

The protein–protein interaction (PPI) network comprising the predicted targets shared between *M. officinalis* compounds and T2DM exhibited significant connections (at a confidence level of 0.7). The network consisted of 134 nodes linked by 398 edges, an average neighbor count of 6.046, a heterogeneity index of 0.874, and a centralization index of 0.195 after excluding isolated nodes, suggesting a moderate degree of connectivity. The median values of the network topological parameters, namely degree, closeness coefficient (CC), and betweenness centrality (BC), were 5, 0.2937 and 0.0291, respectively (Table S5). The top-10 ranking hub genes/T2DM targets (i.e., with degrees higher than twice the median degree of the entire network) were identified as EGFR, SRC, AKT1, TNF, ESR1, HDAC1, CYP3A4, PPARG, PARP1, and MMP9 (Figure 2 and Figure 3).

#### 2.3.2. Compound–Target (CT) Network

**Figure 4:**
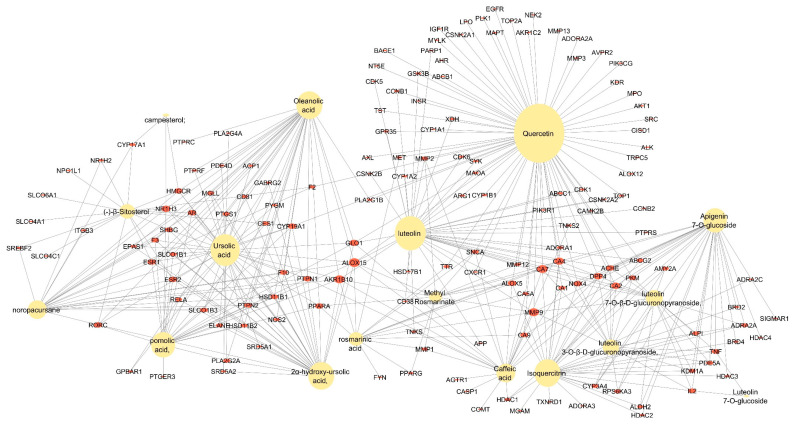
Compound–target (CT) network, with the sizes of the nodes proportional to the magnitude of the degree. Red circles = T2DM targets; yellow circles = *M. officinalis* compounds.

**Figure 5:**
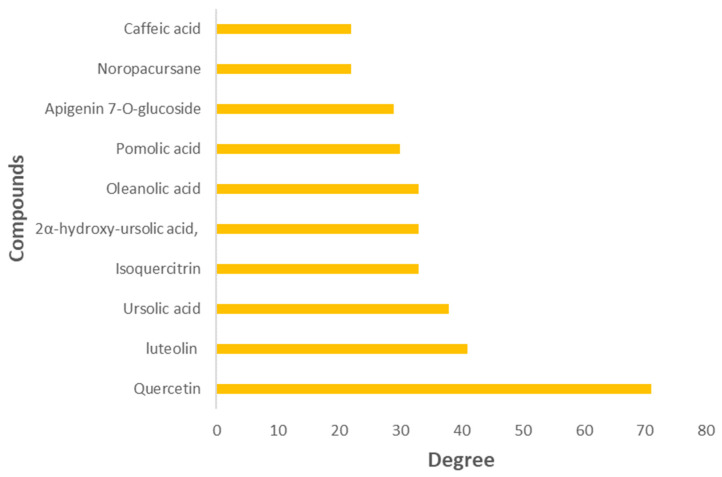
Top-10-ranked (core) *M. officinalis* compounds predicted to interact with T2DM targets.

The compounds associated with the T2DM common targets were linked with the targets in the PPI network above to produce a comprehensive compound–target (CT) network. The composite network comprised 171 nodes (17 compounds and 154 targets); 427 edges; an average number of neighbors of 4.994; and network heterogeneity and centralization of 1.711 and 0.393, respectively. It presented a highly connected structure of multi-targeting compounds and multi-targeted proteins. The key compounds (represented by compound nodes with a high number of connections) were identified based on the ranking of their topological parameters, with several compounds showing a relatively high degree (>4). These included quercetin, luteolin, ursolic acid, isoquercitrin, 2α-hydroxy-ursolic acid, oleanolic acid, pomolic acid, apigenin 7-*O*-glucoside, noropacursane, and caffeic acid, with the first six compounds having degree values greater than 33 (Figure 4 and Figure 5).

#### 2.3.3. Target–Pathway (TP) Network

**Figure 6:**
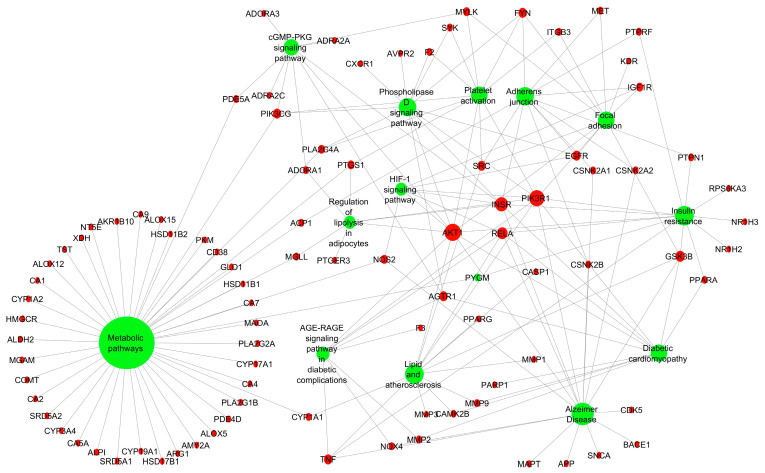
Target–pathway (TP) network of T2DM-relevant pathways enriched by the subset of common targets. Green circles = pathways; red circles = targets.

**Figure 7:**
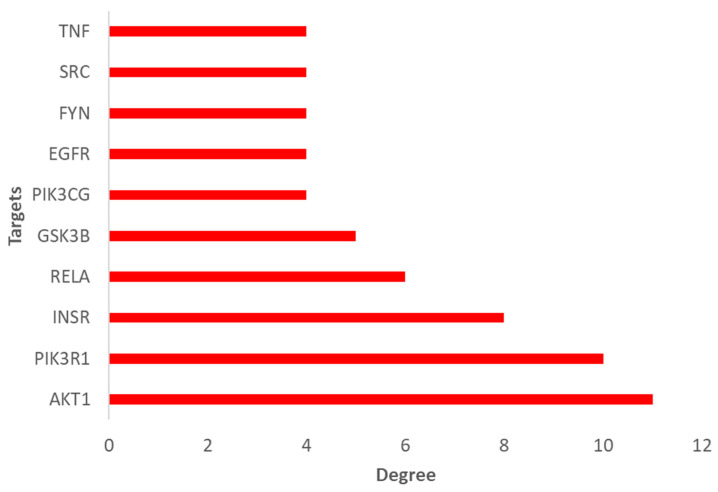
Ranking of top 10 targets enriched in the T2DM pathways.

A pathway enrichment analysis using the predicted 154 common targets was further performed to explore which T2DM-relevant pathways were specifically associated with the top-ranking hub genes/proteins identified in the PPI network. A total of 38 pathways was identified, out of which 12 were specifically linked to T2DM. These included metabolic pathways, insulin resistance, lipid and atherosclerosis, adherens junction, phospholipase D signaling pathway, platelet activation, diabetic cardiomyopathy, focal adhesion, cGMP-PKG signaling pathway, AGE-RAGE (in diabetic complications) signaling pathway, HIF-1 signaling pathway, and regulation of lipolysis in adipocytes (Figure 6). The insulin resistance (Figures S1–S3), adherens junction, and regulation of lipolysis in adipocytes showed highest enrichment folds (Table 1). The top-10 enriched hub targets in the TP network—identified by ranking on degree values—included AKT1, PIK3R1, INSR, RELA, GSK3B, PIK3CG, EGFR, FYN, SRC, and TNF (Figure 7). Interestingly, the list of targets that met the degree selection threshold (value ≥ 2) included proteins such as PTP1N and PPARα, two established targets of existing antidiabetic compounds/drugs [49,50,51] (Table S6).

### 2.4. Gene Ontology and Enrichment Analysis

**Figure 8:**
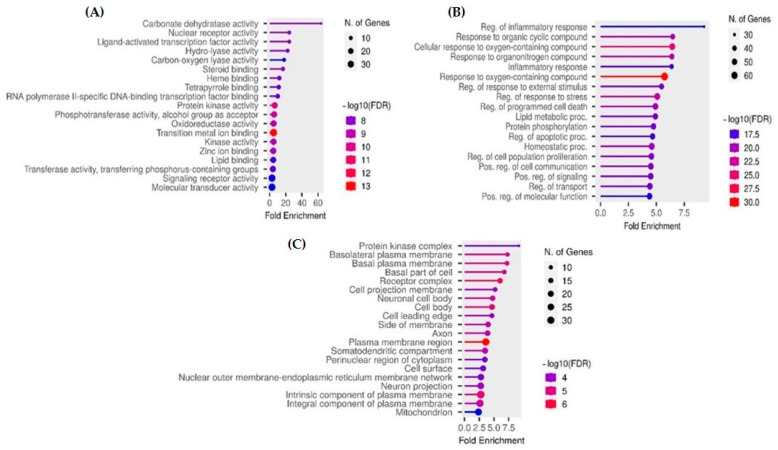
Gene Ontology and KEGG pathway analysis showing the top-ranked enrichments of (**A**) molecular function, (**B**) biological process, and (**C**) cellular compartment.

The enriched molecular functions, biological processes, and cellular components related to the identified common targets are listed in Figure 8A, Figure 8B, and Figure 8C, respectively. This includes molecular functions like nuclear receptor activity, ligand-activated transcription function, hydrolases, kinases, lipid binding, transferase activity, receptor activity, and signal receptor activity. The enriched biological processes included inflammatory process, apoptosis/cell death, lipid metabolism, protein phosphorylation, cell communication, stress response, transport, positive regulation of molecular functions and signaling, and response to organic compounds (including cyclic, oxygen-, and nitrogen-containing compounds). The cellular components included the protein kinase complex, basal/basolateral membrane, components of the plasma membrane, cell projections, axon, cell body, cell surface, neuronal projections, and the nuclear outer membrane–endoplasmic reticulum network.

### 2.5. Molecular Docking

**Figure 9:**
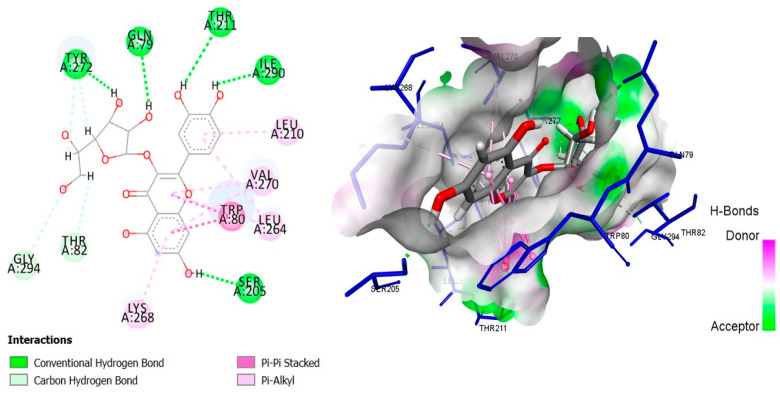
Two- and three-dimensional docked poses of isoquercitrin with AKT1.

**Figure 10:**
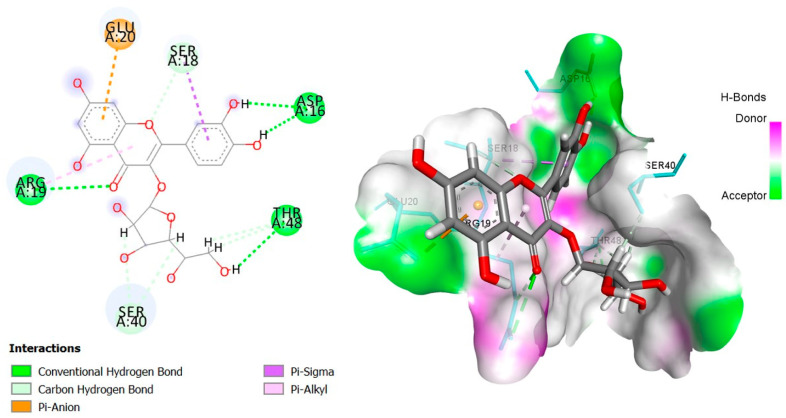
Two- and three-dimensional docked poses of isoquercitrin with PIK3R1.

**Figure 11:**
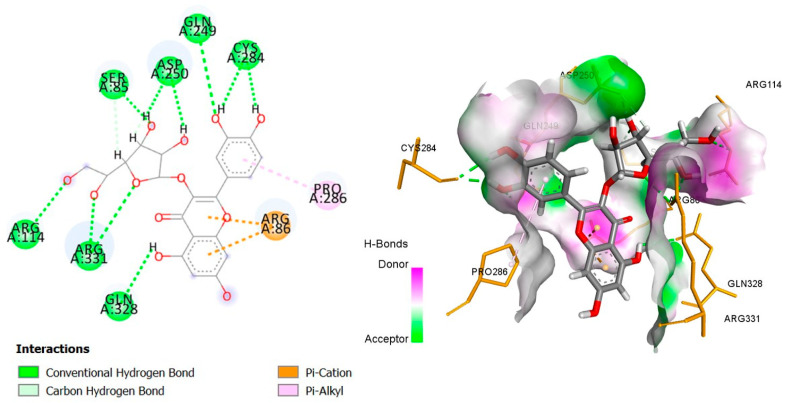
Two- and three-dimensional docked poses of isoquercitrin with INSR.

**Figure 12:**
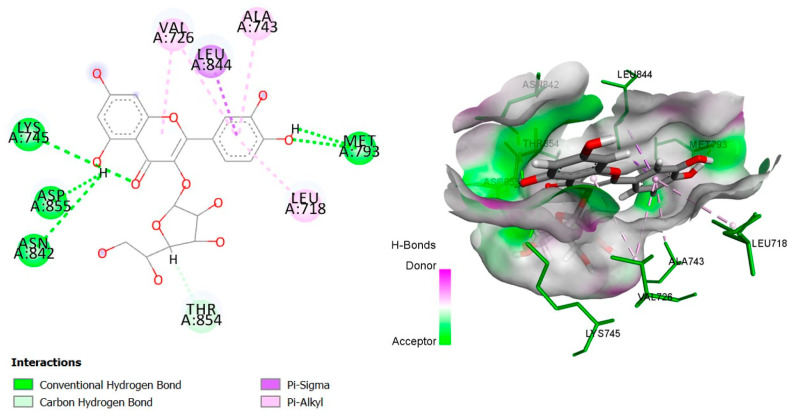
Two- and three-dimensional docked poses of isoquercitrin with EGFR.

**Figure 13:**
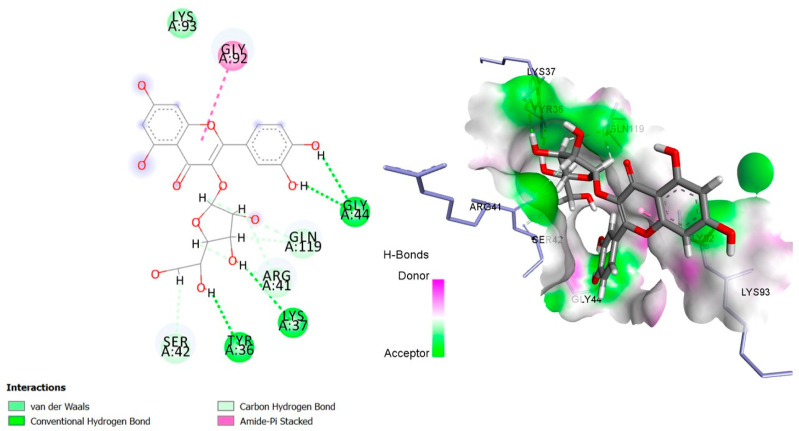
Two- and three-dimensional docked poses of isoquercitrin with RELA (NF-kB p65 subunit).

**Figure 14:**
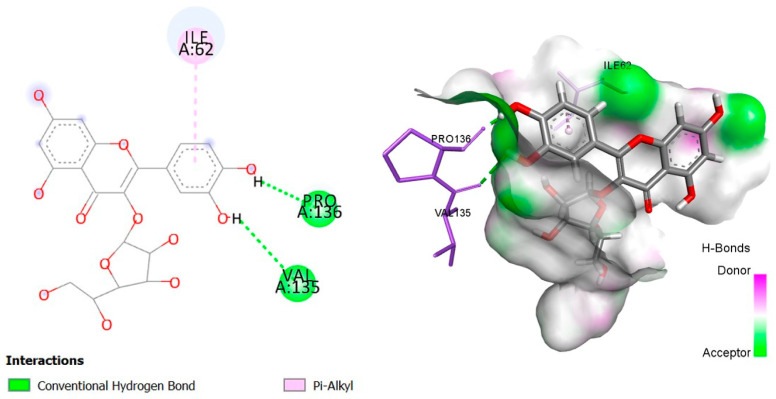
Two- and three-dimensional docked poses of isoquercitrin with GSK3B.

**Figure 15:**
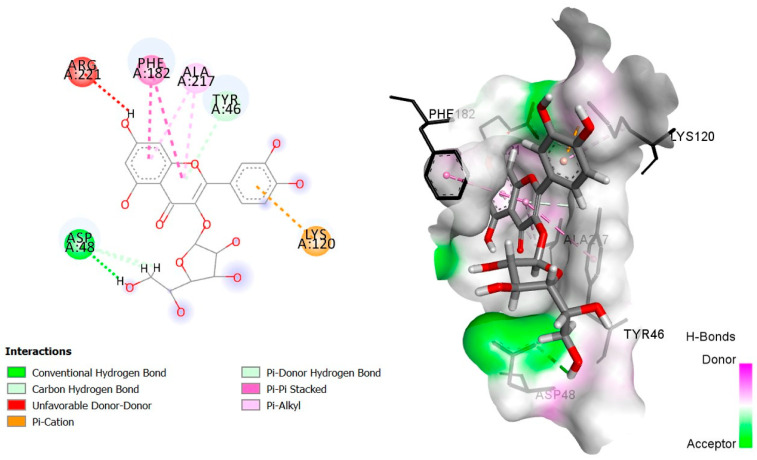
Two- and three-dimensional docked poses of isoquercitrin with PTP1B.

**Figure 16:**
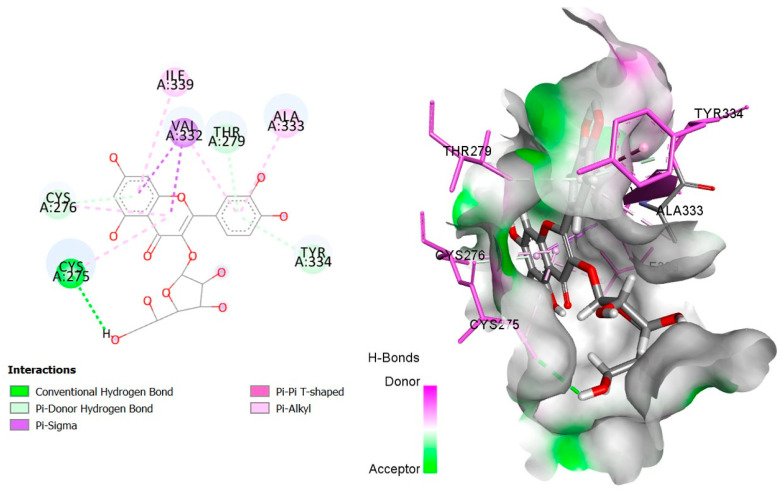
Two- and three-dimensional docked poses of isoquercitrin with PPARα.

The six top-ranked compounds (i.e., quercetin, luteolin, ursolic acid, isoquercitrin, 2α-hydroxy-ursolic acid, and oleanolic acid) predicted in the CT network to be involved in the antidiabetic activity of *M. officinalis* were docked with eight hub targets relevant to T2DM (i.e., AKT1, PIK3R1, INSR, EGFR, RELA (NF-kB p65 subunit), GSK3B, PTP1B, and PPARα). The results indicated that most compounds, especially the flavonoids, exhibited favorable binding energy scores (≤−5 kcal/mol) and SILE values (≥2.0). Isoquercitrin demonstrated the highest docking scores and SILE values when docked with each of the selected targets (Table 2, Table 3, Table 4, Table 5, Table 6, Table 7, Table 8, Table 9, Table 10 and Table 11). The 2D and 3D docked poses between isoquercitrin and each of the tested targets are illustrated in Figure 9, Figure 10, Figure 11, Figure 12, Figure 13, Figure 14, Figure 15 and Figure 16.

### 2.6. Molecular Dynamics (MD) Simulations

The results of the MD simulations are presented in Tables S8–S11. Isoquercitrin showed the most stable binding, with only five targets, namely AKT1, EGFR, INSR, PIK3R1, and PPARα. Among the five systems, the EGFR–ligand complex displayed the lowest average RMSD (0.146 nm), followed closely by AKT1 (0.200 nm). In contrast, the INSR (0.739 nm) and PIK3R1 (0.783 nm) complexes showed significantly larger RMSD values. The PPARα complex exhibited a moderate RMSD of 0.418 nm, indicative of partial stability. These trends were further supported by the RMSF results, with PIK3R1 and EGFR showing the lowest average RMSF values (0.122 and 0.141 nm, respectively). INSR had the highest RMSF (0.498 nm). The lowest Rg value was observed for the PIK3R1–isoquercitrin complex (1.356 nm), followed by EGFR– (1.939 nm) and PPARα–isoquercitrin (1.912 nm). The AKT1–isoquercitrin complex maintained a slightly higher Rg of 2.152 nm, while INSR displayed an Rg of 3.271 nm, aligning with its high RMSD and RMSF values. The SASA values followed a similar trend, with INSR having the highest solvent-accessible surface area (319.790 nm^2^), while PIK3R1 showed the lowest SASA (70.573 nm^2^), consistent with its low Rg and RMSF. The total binding energies, calculated as ΔE = E_complex − (E_receptor + E_ligand), varied across the five complexes, with EGFR showing the most negative binding energy (−30.29 kcal/mol), followed by PPARα (−22.37 kcal/mol) and AKT1 (−19.72 kcal/mol). In contrast, complexes involving PIK3R1 and INSR exhibited higher (less negative) binding energies, measured at −14.30 and −8.77 kcal/mol, respectively.

## 3. Discussion

Protein–protein interaction (PPI) networks are fundamental to understanding the molecular and cellular mechanisms underlying health and disease. Analyzing these sheds light on the processes that initiate and drive disease progression, thereby aiding in the development of effective diagnostic and therapeutic strategies [52,53]. Within these networks, hub genes/proteins play a pivotal role in maintaining network stability and functionality, thereby significantly influencing molecular mechanisms of disease [54,55]. The number of edges in our PPI network (398) was higher than expected, indicating that our protein list exhibits more interactions among its members than would be anticipated for a random set of proteins of the same size and degree distribution drawn from the genome. This suggests that the nodes (proteins) are at least partially biologically connected as a group [56].

Various criteria have been proposed for identifying hub genes within PPI networks. According to the classical network theory, hubs are nodes with a connectivity greater than the average connectivity of the entire network [54,55]. Other studies have defined hubs as nodes with a degree value greater than either the median degree [57,58] or twice the median degree value [39,59,60] of the entire network. The top-10 ranking hubs in the PPI network included proteins previously reported for their potential involvement in the pathogenesis of T2DM, such as EGFR [61,62], SRC [63,64], AKT1 [65,66,67], TNF [68,69], and PPARG [70,71].

The pathways relevant to T2DM, and enriched by our subset of common targets, were predicted to include metabolic pathways, insulin resistance, lipid and atherosclerosis, adherens junction, phospholipase D signaling pathway, platelet activation, diabetic cardiomyopathy, focal adhesion, cGMP-PKG signaling pathway, AGE-RAGE (in diabetic complications) signaling pathway, HIF-1 signaling pathway, and regulation of lipolysis in adipocytes. The enriched hub targets in the TP network included proteins previously reported for their potential involvement in the pathogenesis of T2DM, such as PIK3R1 [72,73], INSR [74], RELA [75,76], GSK3B [77,78], PIK3CG [79,80], and FYN [64,81], and two known target classes of existing antidiabetic compounds/drugs, PTP1N [49] and PPARγ/α [50,51].

Insulin resistance (IR) is a hallmark of T2DM and occurs when there is a suboptimal response to physiological or elevated levels of insulin in insulin-sensitive organs [82]. Al-though the cause and pathogenesis or IR remain unclear, evidence points to several contributing factors. These include defective insulin signaling; defective inter-organ metabolic cross-talk; and dysregulation of metabolic mediators like adiponectin, leptin, and pro-inflammatory cytokines/chemokines (e.g., TNFα, IL-6, and IL-1β). Other factors, such as obesity, genetic mutations, and epigenetic dysregulation, also play significant roles [83]. IR likely arises from any condition that affects insulin signaling by altering the activity or expression of critical targets. Therefore, modulation of these targets is a logical approach to improving IR [84]. The current study identified several key targets involved in insulin signaling within the PPI network, including AKT1, PIK3R1, INSR, and PIK3CG. These targets were enriched in the IR pathway and in other T2DM-relevant pathways.

The PI3K/AKT axis, via the insulin receptor substrate (IRS), plays a central role in linking post-insulin receptor (INSR) signals to downstream targets and effects [85,86]. In diabetes mellitus, these signals can be dysregulated via one or several of the proteins driving insulin signaling (AKT1, PIK3R1, INSR, PPARG, and PIK3CG). Studies have shown that targeted modulation of these proteins can enhance insulin sensitivity. For example, administration of carbenoxolone and catalpol has been found to increase PI3K/AKT expression and improve insulin sensitivity in T2DM mouse models [87,88,89]. Additionally, modulation of PIK3CG and PIK3R1 has shown potential in improving IR [73,90].

Chronic low-grade inflammation is a key feature of obesity and IR in T2DM [88]. This is associated with increased basal level of cytokines (IL-6 and TNF-α) and activation of NF-κB [91,92]. NF-κB activation promotes the production of pro-inflammatory cytokines (IL-6 and TNF-α), which interfere with insulin signaling by upregulating SOCS3 and activating JNK. SOCS3 inhibits insulin receptor substrates, while JNK phosphorylates them, with both actions leading to impaired glucose uptake [93,94]. Inhibition of RELA and TNF, also identified as hub targets in our network, has further been shown to be therapeutically relevant for improving insulin sensitivity and DM [75,95,96].

Glycogen synthase kinase-3 beta (GSK-3β) is a crucial target in insulin signaling with potential applications in T2DM treatment [78]. Overexpression of GSK-3β attenuates insulin signaling by phosphorylating IRS-1 [97]. Studies have also demonstrated that GSK-3β inhibition in animal models stimulates glycogen synthase, improving insulin sensitivity and glucose homeostasis [83,98].

SRC and FYN are members of the Src family of kinases (SFKs), a group of non-receptor tyrosine kinases that regulate a wide range of cellular functions, while EGFR is a member of the ErbB/HER family of receptor tyrosine kinases. SRC is required for glucose utilization under hyperglycemia and mediates glucokinase activity, as well as its subcellular localization [99]. FYN interacts with proteins like nephrin and paxillin during hyperglycemia and impacts cytoskeleton and cell adhesion in podocytes [100]. It also activates other signaling pathways, like EGFR and AKT [101]. EGFR has been reported to maintain β-cell mass in the pancreas [102]. In the context of diabetes pathogenesis, SRC, FYN, and EGFR are significantly implicated in the development of vascular complications, such as diabetic kidney injury, and have been reported to prevent/improve diabetic nephropathy and other diabetic-related kidney injuries [63,64,103]. They have also been reported to improve insulin sensitivity in a diabetic nephropathy mouse model [104].

PPARA (PPARα) and PPARG (PPARγ) are two isoforms of the peroxisome proliferator-activated receptors, and are members of the nuclear hormone receptor superfamily [105]. They play critical roles in the regulation of lipid and glucose metabolism and are dysregulated in T2DM. PPARA is mainly expressed in metabolically active tissues (e.g., liver, kidney, skeletal muscle, and brown adipose tissue) and is involved in regulating lipid metabolism and inflammation. PPARG is highly expressed in the colon, immune cells, and white adipose tissue. It is crucial for adipose tissue development and maintenance, and it serves as a central regulator of glucose homeostasis, insulin signaling, and insulin sensitivity [106]. PPAR agonists activate PPARA and/or PPARG receptors, improving inflammation, insulin sensitivity, hyperglycemia, and hyperlipidemia, which are the hallmarks of T2DM [107].

PTP1N (PTP1B) is a negative regulator of insulin signal transduction. Inhibition or modulation of PTP1B improves insulin sensitivity, glycemic control, and resistance to diet-induced obesity [108,109,110].

Overall, targets directly implicated in the insulin signaling pathway/IR are most critical to the pathophysiology of T2DM. In this study, these include AKT1, PIK3R1, INSR, and PPARG, which are direct components of the pathway, and RELA, GSK3B, PTP1B, PPARA, which are also targeted to enhance insulin signaling. SRC/EGFR/FYN, on the other hand, provides some therapeutic leverage, but it more specifically addresses diabetes-related complications.

Most of the highlighted targets aforementioned are linked with the IR pathway ( Figure 5). Other T2DM-relevant pathways which showed notable enrichment in the TP network included adherens junctions, regulation of lipolysis in adipocytes, platelet activation, and phospholipase D signaling pathways. Adherens junctions provide crucial adhesive contact support between neighboring endothelial cells [111]. In pancreatic β cells, cadherin-mediated adherens junctions are involved in insulin secretion and may be disrupted by hyperglycemia, contributing to diabetic complications [112,113]. This pathway is mediated and regulated by kinases (CSNK2A1), tyrosine phosphatases (PTPRF, PTGS2), and β-catenin (CTNNB1) [114].

The relationship between lipolysis and IR is well known. Dysregulation of basal fat cells has been reported to be connected to IR. Studies in mouse models have shown that inhibition of fat cell lipase improves whole-body insulin sensitivity and glucose metabolism [115].

Increased platelet reactivity or activation is a common feature in people with DM, and it is exacerbated by insulin resistance [116]. This phenomenon is a major factor contributing to cardiovascular disease (CVD) risk in DM by enhancing platelet adhesion and aggregation [117].

Phospholipase D (PLD) signaling is implicated in glucose-induced insulin secretion and diabetic retinopathy [118,119]. It also plays a critical role downstream of insulin signaling. Activation of PLD promotes glucose uptake and glycogen synthesis in insulin-sensitive cells [120,121,122].

Other pathways associated with T2DM (and its complications) identified in this study were AGE-RAGE, lipid atherosclerosis, diabetic cardiomyopathy, HIF-1 signaling, cGMP-PKG, focal adhesion, and various metabolic pathways. An initial poor glycemic control is associated with diabetic complications [123]. Prolonged hyperglycemia stimulates the formation of hyperglycemia-derived substances known as advanced glycation end-products (AGEs) [124], which, in turn, accelerate the expression of receptor for advanced glycation end-product (RAGE). AGE binds to RAGE to trigger intracellular signals that culminate in ROS production, induction of oxidative and endoplasmic reticulum stress, intracellular glycation, cellular damage, activation of pro-inflammatory activity, and ultimately diabetic vascular complications. AGEs have also been suggested to act as a metabolic memory for the progression of chronic diabetic complications [125]. Therefore, the inhibition of AGE-RAGE formation is a therapeutic approach that has the potential to ameliorate DM and its complication. One study has reported that pyridoxamine could limit the production of AGE in a streptozocin-induced diabetic rat model [126].

The cGMP-dependent PKG pathway is involved in a wide range of downstream effects, such as vasodilation, relaxation, proliferation, and apoptosis [127]. Studies have shown that enhancement of the cGMP-dependent pathway activity improves hyperglycemia [128,129,130].

The compound–target (CT) network was constructed to obtain a comprehensive overview of the *M. officinalis* compounds interacting with the molecular targets predicted to be implicated in T2DM. Quercetin, luteolin, ursolic acid, isoquercitrin, 2α-hydroxy- ursolic acid, and oleanolic acid were identified as the top-ranking compounds in the CT network, exhibiting a high degree of connections (degree value ≥ 33) to various T2DM-relevant targets. This indicated that these compounds—which belong to the class of flavonoids and pentacyclic triterpenoids—exerted their effects via a multimodal mechanism of action. The antidiabetic activity of flavonoids is well-documented with studies previously reported on isoquercitrin [131,132,133,134], quercetin [135,136,137], and luteolin [138,139]. Isoquercitrin can activate AMP-activated protein kinase (AMPK) [140,141], modulate galectin-3-mediated IR and lipid metabolism [142], and ameliorate insulin sensitivity in Ring finger protein 6 (RNF6) overexpression db/db mouse models [143]. This flavonoid has also been reported to suppress pro-inflammatory cytokines and mediators in LPS-activated mouse models [144,145,146]; increase the expression of PKA, AKT, INSR, and PIK3R1; and inhibit DPP4 [132] and PTP1B in vitro [147]. Quercetin and luteolin have been reported to improve glucose uptake and insulin sensitivity in skeletal muscle cells (H6 myotubes) and endothelium models, respectively [148,149,150,151]. They can inhibit GSK-3 [152,153], ameliorate oxidative stress, and reduce the formation of AGE products [154,155]. Luteolin has been reported to inhibit pro-inflammatory targets and mediators (NF-kB, TNFα, and IL-6) in different experimental models, in turn improving insulin sensitivity and IRS/AKT expression [149]. Quercetin has been reported to reduce the transcriptional activity of NF-κB in stable coronary artery disease [156]. Ursolic acid and some synthetic derivatives of ursolic acid have previously demonstrated antidiabetic activity by inhibiting alpha-glucosidase activity in vitro and lowering blood glucose in vivo [157,158]. The antihyperglycemic effect of ursolic acid has been related to insulin secretion and the insulinomimetic effect on glucose uptake via increased synthesis and translocation of GLUT4 [157]. Other studies have revealed that ursolic acid, oleanolic acid, and other related pentacyclic triterpenes can improve DM by activating the insulin signaling pathway [159,160,161] and increasing insulin sensitivity via the IRS/PI3K/AKT signaling pathways in 3T3-L1 adipocytes [162,163,164,165]. They also show significant inhibition of PTP1B both competitively and allosterically [166,167].

Molecular docking is commonly used for modeling and characterizing the behavior of small molecules within the binding sites of target proteins in an effort to understand fundamental biochemical processes at the molecular level [168,169]. It was used in the current study to investigate the binding affinity and interactions of *M. officinalis* compounds with hub targets enriched in the identified T2DM-relevant pathways. The results showed that quercetin, luteolin, ursolic acid, isoquercitrin, 2α-hydroxy-ursolic acid, and oleanolic acid had relatively high binding scores and SILE values, with flavonoids—particularly isoquercitrin—exhibiting the highest SILE values for most of the targets screened. Isoquercitrin showed various interactions with the protein targets, including interactions with key amino acid residues at the binding sites. These included hydrogen bonds with Asp48 and hydrophobic interactions with Lys120, Tyr46, Phe182, and Ala217 for PTP1B [170,171]; hydrogen bonds with Lys37, Tyr36, Gly44, Arg41, Gln119, and Ser42 for RELA (NF-κB p65 subunit) [172]; hydrogen bonds with Cys275, Cys276, Thr279, and Tyr334, and hydrophobic interactions with Val332, Tyr334, Cys275, Cys276, Ile339, and Ala333 for PPARα [173]; hydrogen bonds with Ser85, Arg114, Gln249, Arg331, Asp250, Gln328, and Cys284, and hydrophobic interactions with Arg86 and Pro286 for INSR [174,175]; hydrogen bonds with Tyr272, Gln79, Ser205, Thr211, and Ile290, and hydrophobic interactions with Gly294, Tyr272, Thr82, Trp80, Leu264, Lys268, Val270, and Leu210 for AKT1 [176]; and hydrogen bonds with Val135 and Pro136, and hydrophobic interactions with Ile62 for GSK-3β [177]. In PI3KR1, the hydrogen bonds were with amino acid residues within the SH3 domain of the regulatory protein subunit. The phenotypes of mutations in different regions of the PI3KR1 gene are quite diverse, making it difficult to target [178]. With more specific mutation–phenotype evidence, targeting PI3KR1 for DM treatment could become more attractive [179]. To date, direct inhibitors of PI3KR1 are still lacking, despite the logical premise that controlled modulation of PI3KR1’s inhibitory action on the catalytic subunit of PI3K, PIK3CA, could be beneficial.

MD studies were further conducted to gain detailed insights into the conformational dynamics and compactness of each isoquercitrin–protein complex under simulated physiological conditions [180]. The low RMSD values obtained for the EGFR–isoquercitrin and the AKT1–isoquercitrin complexes indicated low conformational fluctuations and suggested stable binding [181], unlike the INSR– and PIK3R1–isoquercitrin complexes, which had notable conformational fluctuations (high RMSD), reflecting unstable interactions or incomplete binding. PIK3R1 and EGFR showed the lowest average RMSF values (a measure of the local flexibility of residues), implying a well-maintained local structural integrity [182], unlike INSR (highest RMSF), which indicated substantial mobility in specific regions that compromised stable ligand association. The low Rg values obtained for the PIK3R1–isoquercitrin complex, followed by the EGFR– and PPARα–isoquercitrin complexes, indicated these had compact and tightly folded structures [183], while the INSR-isoquercitrin complex (highest Rg value) suggested structural relaxation or destabilization, in alignment with its high RMSD and RMSF values. PIK3R1 showed the lowest SASA value, reflecting a more folded and less exposed state [184], while INSR (highest SASA value) had a more unfolded or exposed state.

MM-PBSA calculations were performed to gain deeper insight into the binding free energies governing the interaction between the selected compounds and the respective protein targets, thus offering a thermodynamic perspective on the stability and affinity of the ligand–protein complexes [185,186]. EGFR showed the most negative binding energy, followed by PPARα and AKT1. These values suggest that isoquercitrin formed more energetically favorable and stable complexes with these targets. The notably more negative energy for EGFR, in particular, implies stronger intermolecular interactions that are most likely dominated by van der Waals forces and hydrophobic interactions, which contribute significantly to the stabilization of the complexes. The relatively less favorable binding energies (less negative) observed for the PIK3R1– and INSR–isoquercitrin complexes indicate that weaker protein–ligand interactions and reduced stability, which could reflect suboptimal ligand orientation, limited contact surface within the binding pocket, or the presence of repulsive electrostatic interactions. This difference in binding energy clearly emphasizes the superior potential of EGFR, PPARα, and AKT1 as target proteins for the tested compounds, due to their capacity to form more stable and thermodynamically favorable interactions.

## 4. Limitation of the Study and Conclusions

This study aimed to predict the molecular targets and mechanisms of action of *M. officinalis* phytoconstituents in T2DM using compounds retrieved from literature mining. Only constituents that were fully and unambiguously characterized by spectrometric/spectroscopic techniques were selected for the in silico analyses. Whilst literature mining is often used for the selection of phytoconstituents in network pharmacology and other in silico studies [41,187], such an approach without any direct experimental validation only highlights putative biological targets/active compounds involved in the studied disease condition [188]. As such, the results should be interpreted as exploratory insights rather than definitive proof, since computational predictions cannot fully capture biological complexity, pharmacokinetics, bioavailability, or potential toxicity. Furthermore, the analysis was restricted to compounds already reported in the literature, meaning that other bioactive constituents may have been overlooked.

In this study, network pharmacology analysis predicted that multiple compounds from *M. officinalis* aerial parts—primarily flavonoids and pentacyclic triterpenoids—could modulate various targets and pathways related to T2DM. Molecular docking, followed by MD studies, identified that the flavonoid isoquercitrin showed energetically favorable and stable complexes with three protein targets involved in T2DM, namely EGFR, PPARα, and AKT1. Interestingly, isoquercitrin has previously demonstrated promising antidiabetic activity in vitro and in vivo [131,132,133,134,140,141,142,143,144,145,146,147], thus supporting the computational predictions obtained here. Nevertheless, docking and simulation data only indicate potential binding interactions and do not account for other pharmacological factors or the multi-component nature of plant extracts, where synergistic or antagonistic effects may play a major role.

Additional experimental work is warranted to confirm the effect of isoquercitrin on the three aforementioned targets. In particular, enzyme-based assays, cellular models, and in vivo studies are needed to validate the computational outcomes and clarify the pharmacological profile of isoquercitrin. Pharmacokinetic and safety studies would also be crucial to assess its translational value. Further clinical studies, exploring both therapeutic efficacy and safety, may eventually support the development of isoquercitrin-based *M. officinalis*-derived antidiabetic therapies.

## 5. Materials and Methods

### 5.1. Compounds Mining

The SciFinder database (https://scifinder.cas.org, accessed on 7 January 2024) was used to choose compounds based on a literature search using the keywords (“Melissa officinalis” OR “M. officinalis”) AND “NMR”; and (“Melissa officinalis” OR “M. officinalis”) AND “Nuclear Magnetic Resonance” to retrieve 31 research articles. Compounds were chosen only from research articles that explicitly reported the purification of phytochemicals from the aerial parts—leaves and stems—of *Melissa officinalis* L. and their unambiguous characterization using NMR. The canonical smiles of the selected compounds were obtained from SciFinder, PubChem (https://pubchem.ncbi.nlm.nih.gov/, accessed on 12 January 2024), or Chemspider (http://www.chemspider.com, accessed on 12 January 2024) databases. For compounds not found in these databases, ACD Chemschetch (v. 12.0) was used to draw the compound structures and generate their canonical SMILES. An Excel database file was created to store all the obtained compounds, along with their canonical SMILES.

### 5.2. Ranking of Mined Compounds

The SwissADME tool (http://www.swissadme.ch, accessed on 20 January 2024) was used to compute the physicochemical (molecular weight, lipophilicity, water solubility, rotatable bonds, and H-bond donors/acceptors), pharmacokinetics (GI absorption, blood–brain barrier penetration, and P-glycoprotein substrate inhibition), and drug-likeness properties (by filtering for violations of Lipinski/Veber/Egan/Ghose/Muegge rules and assessing the oral Abbott bioavailability score) of the retrieved compounds [189]. The drug likeness scores were computed using the online Molsoft LLC drug-likeness and molecular property predictor (https://molsoft.com/mprop/, accessed on 27 January 2024). A two- filter approach was implemented using the oral Abbott bioavailability score (≥0.5) [190] and the drug-likeness score (>0.18) [191]. Twenty-one compounds were selected for further analysis, as they met at least one of these two conditions.

### 5.3. Compound- and Disease-Associated Targets

The Similarity Ensemble Approach (SEA; https://sea.bkslab.org/, accessed on 28–30 January 2024), Swiss Target Prediction (STP; (http://www.swisstargetprediction.ch/, accessed on 28–30 January 2024), and BindingDB (https://www.bindingdb.org/, accessed on 28–30 January 2024) online servers were used to predict the gene/protein targets associated with the *M. officinalis* compounds. Only targets in the highest-ranking category (i.e., with a score ≥ 0.60) were selected to ensure a high confidence prediction [192,193,194,195,196]. To identify and eliminate duplication, the predicted targets were standardized across all three databases using their official gene symbols. Duplicates from the three databases appeared as common/shared targets and were merged into one using the online tool VENNY 2.1 (https://bioinfogp.cnb.csic.es/tools/venny/, accessed on 18 February 2024). Following on from that, the entire collection of compound-associated targets was further filtered using Microsoft Excel to remove redundancies resulting from compounds with the same targets and obtain a final unique set of compound-associated targets.

For the disease-associated protein targets linked to T2DM, various online databases, including GeneCards (https://www.genecards.org/, accessed on 2 February 2024), TTD (https://idrblab.net/ttd/, accessed on 2 February 2024), DisGeNET (https://www.disgenet.org/, accessed on 4 February 2024), KEGG (https://www.genome.jp/entry/H00409, accessed on 4 February 2024), and Malacards (https://www.malacards.org/, accessed on 8 February 2024) were queried. The search terms consisted of “non-insulin dependent diabetes mellitus” (for DisGeNET exclusively) and “type-2 diabetes mellitus” (for all other databases). The resulting compound-associated targets and disease-associated targets were merged to extract a shared subset of targets using VENNY 2.1 (accessed on 18 February 2024). These shared targets were then used to construct the network.

### 5.4. Network Construction and Analyses

The different networks, including the protein–protein interaction (PPI), compound–target (CT), and target–pathway (TP) networks, were constructed using Cytoscape v.3.9.1, following a previous methodology with slight modifications [190].

#### 5.4.1. Protein–Protein Interaction (PPI) Network

The subset of common targets was uploaded onto the STRING online tool (https://string-db.org/, accessed on 18–19 February 2024) to predict the PPI network. The minimum required interaction was set at a high confidence level (score of 0.7), and the organism was set to *Homo sapiens*. The predicted network was exported to Cytoscape for further analysis. The CytoHubba plugin app was downloaded and activated in Cytoscape to rank and identify the core targets within the PPI network. The relevant topology parameters, such as degree, betweenness centrality, closeness centrality, and stress, were computed using the “Analyse network” option in Cytoscape.

#### 5.4.2. Compound–Target (CT) Network

The identified compounds and their associated common targets were organized into a database in Excel. The predicted network was exported to Cytoscape using the CytoHubba plugin app, as aforementioned, to rank and identify core compounds linked with the targets in the PPI network. The default “Analyse network” option was used to determine the relevant topology parameters as above.

#### 5.4.3. Target–Pathway (TP) Network

The common targets were uploaded onto the Database for Annotation, Visualization, and Integrated Discovery (DAVID; https://david.ncifcrf.gov/tools.jsp, accessed on 22 February 2024) to retrieve the key pathways linked to these proteins. The pathways with *p*-value ≤ 0.05, false discovery rate (FDR) ≤ 0.05, and then Benjamini value < 0.05 were selected [190,197] and tabulated for further work. The pathways directly linked with T2DM were identified and used for the TP construction in Cytoscape to help visualize the connections between targets and associated pathways. The most enriched pathways relevant to T2DM were visualized and presented using the ShinyGO program v. 0.8 (http://bioinformatics.sdstate.edu/go/, accessed on 24 February 2024) for illustrative purposes.

### 5.5. Gene Ontology and KEGG Pathway Enrichment Analysis

The cellular component, biological process, molecular function, and KEGG pathways associated with the common targets were identified using the ShinyGO program v. 0.8. The standardized gene symbols of each target were uploaded to ShinyGO, specifying the species as *Homo sapiens* and setting the FDR cut-off value at 0.05 [198]. Pathways were selected by FDR (*p* < 0.05) and sorted by fold enrichment.

### 5.6. Molecular Docking

The protocol used for the molecular docking study was based on a previous method [41].

#### 5.6.1. Protein Target Preparation

The X-ray crystallography structural data of key targets were downloaded in .*pdb* format with their co-crystallized ligand (serving as control) from the RCSB PDB database (https://www.rcsb.org/ accessed on 25 February 2024). This included targets associated prominently with T2DM pathways from the TP network, namely AKT1 (PDBID: 3O96), PIK3R1 (PDB ID: 2IUG), INSR (PDB ID: 6PXW), EGFR (PDB ID: 2RGP), RELA (NF-kB p65 subunit) (PDB ID: 1NFI), and GSK3B (PDB ID: 4AFJ), as well as two well-established T2DM targets, namely PTP1B (PDB ID: 4Y14) and PPARα (PDB ID: 6LXC), identified within the PPI and TP networks.

Protein target preparation was performed using the tools and protocols available in MOE 2015. For each protein, the chain A was selected for computation. Heteroatoms, including water, were deleted via the Sequence (*seq*) editor in MOE. The QuickPrep function in MOE was used to prepare the protein targets: This process involved protein structure validation and correction followed by protonation in 3D under physiological conditions (T = 310 K, pH = 7.4, and salt-concentration = 0.1 mol/L). Partial charges were applied using the “adjust hydrogen and lone pair as required” option, and the entire system was then energy-minimized using the Amber10:EHT force field. The fully prepared and optimized 3D protein structures were saved in *.moe* format for subsequent docking.

#### 5.6.2. Binding/Docking Site Prediction

The docking sites were determined based on the binding sites of the respective co-crystallized ligands for targets with co-crystallized ligands. The SiteFinder program in MOE was used to predict the active binding sites for targets without co-crystallized ligands.

#### 5.6.3. Ligand Preparation

The top-ranked compounds from the aerial parts of *M. offcinalis* L., namely quercetin [199], luteolin [200], isoquercitrin [201], ursolic acid, 2α-hydroxy-ursolic acid, and oleanolic acid [202], predicted in the CT network as the key compounds involved in the antidiabetic activity of this plant, were selected as the ligands for the molecular docking study. The co-crystallized ligand of each protein was isolated and used as a reference control, except for RELA, INSR, and PIK3R1 (which lacked co-crystallized ligands). For these targets, dehydroxymethylepoxyquinomicin, metformin, and wortmannin were employed as reference controls, respectively. The structures of these compounds were downloaded in SDF format from PubChem and imported into MOE, and a compound library was prepared in .*mdb* format. The preparation protocol involved several key steps to ensure that all compounds were in the correct chemical and conformational state for downstream molecular docking analysis: each compound was subjected to a standardization protocol using the WASH function in MOE. This corrected valency, adjusted tautomeric and ionization states, ensured apt protonation at pH 7.4, and added/removed explicit hydrogens as required. Following washing, partial atomic charges were calculated and assigned. Subsequently, the structures were subjected to energy minimization using steepest descent/conjugate gradient algorithms with the Root Mean Square (RMS) gradient set at 0.1 kcal/mol/Å to ensure low energy confirmation for each ligand. All computation was performed using the using the Amber10:EHT force field. The prepared and optimized compound structures were then saved in .*mdb* format, ready for use in molecular docking studies.

#### 5.6.4. Validation of Docking

A dummy docking was carried out using the X-ray crystallography structures of the target proteins and their co-crystallized ligands. Each isolated co-crystallized ligand was re-docked onto the binding site of its corresponding target protein. This was repeated with a different scoring function—ASE, Affinity dG, Alpha HB, Electron Density, GBVI/WSA dG, and London dG/—each time. The docked binding pose for each scoring function was compared to the experimentally determined pose in the X-ray complex in Drug Discovery studio. An RMSD value ≤ 2.0 Å (relative to the native binding pose of the control ligand) is considered a good validation of the docking results [203]. While several scoring functions produced RMSD values close to or slightly above this threshold, the London dG/GBVI/WSA dG scoring function yielded the lowest RMSD and was therefore selected for the final docking, combined with the induced fit refinement method (see Table S7).

#### 5.6.5. Docking Simulations

Docking simulations were performed on MOE following a previously reported protocol [41]. The MOE-Dock protocol comprised four key stages: generation of ligand conformations; optional pharmacophore-based filtering; ligand placement and scoring within the binding pocket; and flexible refinement of the receptor–ligand complex, followed by re-scoring. For this study, all ligands were docked using the triangular matcher placement/flex receptor refinement methods (default in MOE) [204]. The poses generated by the placement method were scored using the London dG scoring function and subsequently re-scored using GBVI/WSA dG [204,205]. The simulation was performed on an Intel core i7 CPU @ 2.00 GHz, 2.60 GHz system under the Amber10:EHT force field. The docked poses and their respective scores were saved as outputs in .*mdb* format in a database. The protein–ligand complexes were visualized (2D and 3D) for interactions with active site amino acid residues using Drug Discovery Studio v. 16.0. The docking scores of the top-ranking poses for each of the selected ligands were normalized by computing the corresponding size-independent ligand efficiency values (SILEs) [206].

### 5.7. Molecular Dynamics Studies

Molecular dynamics (MD) simulations were carried out for 100 nanoseconds using GROMACS 2023 with the CHARMM36 force field to ensure accurate modeling of molecular interactions [207]. Ligand topologies were generated using SwissParam, facilitating smooth integration into the simulation workflow [208]. Each complex was embedded in a dodecahedral simulation box, solvated using the TIP3P water model, and neutralized with counter-ions. Energy minimization was performed using the steepest descent algorithm until the system reached a maximum force threshold below 10.0 kJ/mol. Subsequently, NVT equilibration was conducted for 500 ps at 300 K using a V-rescale thermostat, followed by NPT equilibration for 100 ps under Berendsen pressure coupling with a coupling constant of 2.0 ps [209,210]. Several key descriptors were analyzed to evaluate the dynamic behavior and structural integrity of each ligand–protein complex. These included the Root Mean Square Deviation (RMSD) to track conformational stability over time, Root Mean Square Fluctuation (RMSF) to assess residue-level flexibility, Radius of Gyration (Rg) to determine protein compactness, and Solvent Accessible Surface Area (SASA) to evaluate solvent exposure. To complement the structural analysis, an MM-PBSA approach was also employed to estimate the binding free energies of each complex [185,186].

## Figures and Tables

**Table 1:** Pathways enriched by the subset of common targets, with T2DM-relevant pathways highlighted in bold. * The most highly enriched pathways.

Pathways Enriched	Gene Count	Fold Enrichment
**Metabolic pathways**	**45**	**1.79**
Pathways in cancer	26	3.00
Alzheimer disease	16	2.56
Chemical carcinogenesis—receptor activation	15	4.34
Chemical carcinogenesis—reactive oxygen species	15	4.13
Steroid hormone biosynthesis	13	12.86
**Insulin resistance**	**13**	7.38 *
Neutrophil extracellular trap formation	13	4.17
Proteoglycans in cancer	13	3.89
**Lipid and atherosclerosis**	**13**	**3.71**
**Adherens junction**	**12**	7.91 *
**Phospholipase D signaling pathway**	**12**	**4.97**
**Platelet activation**	11	**5.44**
**Diabetic cardiomyopathy**	**11**	**3.32**
**Focal adhesion**	11	**3.32**
Viral carcinogenesis	11	3.31
Prostate cancer	10	6.32
Relaxin signaling pathway	10	4.75
Hepatitis C	10	3.88
**cGMP-PKG signaling pathway**	**10**	**3.67**
Ovarian steroidogenesis	9	10.82
EGFR tyrosine kinase inhibitor resistance	9	6.99
Endocrine resistance	9	5.63
Thyroid hormone signaling pathway	9	4.56
Yersinia infection	9	4.03
Fluid shear stress and atherosclerosis	9	3.97
Prolactin signaling pathway	8	7.01
PD-L1 expression and PD-1 checkpoint pathway in cancer	8	5.51
**AGE-RAGE signaling pathway in diabetic complications**	**8**	**4.91**
Progesterone-mediated oocyte maturation	8	4.81
C-type lectin receptor signaling pathway	8	4.72
**HIF-1 signaling pathway**	**8**	**4.50**
**Regulation of lipolysis in adipocytes**	**7**	7.40 *
Arachidonic acid metabolism	7	7.04
Fc epsilon RI signaling pathway	7	6.31
Nitrogen metabolism	6	21.65
Linoleic acid metabolism	6	12.27
Antifolate resistance	5	10.22

**Table 2:** Docking binding energy scores (in kcal/mol), SILE values, and interacting residues of key *M. officinalis* compounds docked with RAC-alpha serine/threonine-protein kinase (AKT1).

Compound	Docking Score	SILE Value	Interacting Amino Acid Residues
**Isoquercitrin**	−8.5301	2.99	(TYR272, GLN79, SER205, THR211, ILE290)a, (GLY294, TYR272, THR82)b, TRP80g, (LEU264, LYS268, VAL270, LEU210, LEU264)e
**Luteolin**	−6.3282	2.54	(SER205, THR211)a, ASP292c, (LEU264, TRP80)g, (LEU210, LYS268, VAL270)e
**Quercetin**	−6.6767	2.64	(THR211, ILE290)a, ASP292c, TRP80g, (LEU264, LYS268, VAL270, LEU210)e
**Ursolic acid**	−6.8246	2.39	ARG273a, (LEU264, VAL270, ILE84, TRP80)e
**2β-hydroxy-ursolic acid**	−6.6854	2.32	TRP80g, (VAL270, TRP80)e
**Oleanolic acid**	−6.7658	2.37	TYR272b, (VAL270, ILE84)e
**PubChem ID: 10196499 (Control)**	−10.1961	3.32	SER205a, (ASP274, ARG273, ASP292)c, (ILE84, TRP80)g, (LEU264, VAL270, LYS268, LEU210)e

a = conventional H-bond, b = carbon–H-bond, c = electrostatic (Pi-cation), e = alkyl/alkyl Pi, g = Pi-sigma, Pi-Pi-stacking, Pi-Pi-T-shaped.

**Table 3:** Docking binding energy scores (in kcal/mol), SILE values, and interacting residues of key *M. officinalis* compounds docked with phosphatidylinositol 3-kinase regulatory subunit alpha (PIK3R1).

Compound	Docking Score	SILE Value	Interacting Amino Acid Residues
**Isoquercitrin**	−6.538	2.29	(ARG19, THR48, ASP16)a, (SER18, SER40, THR48)b, GLU20c, (SER18, ARG19)g
**Luteolin**	−5.2333	2.10	(ARG19, HIS44)a, (ARG37, SER40)d, ARG19e
**Quercetin**	−4.7769	1.89	(ARG19, THR48)a,(ARG37, SER40)d, ARG19e
**Ursolic acid**	−4.8283	1.69	LYS61B, HIS44g, (LEU59, HIS44)e
**2β-hydroxy-ursolic acid**	−5.3987	1.87	(ARG37, THR48)a, LEU59e
**Oleanolic acid**	−4.9497	1.73	(SER40, THR41)a, (ARG19, LEU59, HIS44)e
**PubChem ID: 312145** **(Control)**	−5.4397	1.94	SER40a, (SER40, THR48)b, HIS44g

a = conventional H-bond, b = carbon–H-bond, c = electrostatic (Pi-cation), d = H-bond (Pi-donor/acceptor), e = alkyl/alkyl Pi, g = Pi-sigma, Pi-Pi-stacking, Pi-Pi-T-shaped.

**Table 4:** Docking binding energy scores (in kcal/mol), SILE values and interacting residues of key *M. officinalis* compounds docked with the insulin receptor (INSR).

Compound	Docking Score	SILE Value	Interacting Amino Acid Residues
**Isoquercitrin**	−6.868	**2.41**	(SER85, ARG114, GLN249, ARG331, ASP250, GLN328, CYS284)a, ARG86c, PRO286e
**Luteolin**	−5.913	2.37	(GLN249, CYS284, SER85)a, PRO286b, ARG331c
**Quercetin**	−5.928	2.35	(GLN249, ASP250, CYS284, SER85)a, PRO286b, ARG331c
**Ursolic acid**	−6.543	2.29	LYS283, ARG331)a, (PRO286, ARG331 ILE285, LYS283)e
**2β-hydroxy-ursolic acid**	−6.941	2.41	(LYS283, PRO286, ARG331, ILE285)e
**Oleanolic acid**	−6.441	2.26	(ARG86, GLN328)a, (PRO286, HIS275)e
**PubChem ID: 445421 (Control)**	−4.521	2.34	(CYS284, HIS247)a

a = conventional H-bond, b = carbon–H-bond, c = electrostatic (Pi-cation), e = alkyl/alkyl Pi, Pi-Pi-stacking, Pi-Pi-T-shaped.

**Table 5:** Docking binding energy scores (in kcal/mol), SILE values, and interacting residues of key *M. officinalis* compounds docked with the epidermal growth factor receptor (EGFR).

Compound	Docking Score	Sile Value	Interacting Amino Acid Residues
**Isoquercitrin**	−8.1937	**2.87**	(LYS745, MET793, ASN842, ASP855, MET793)a, THR854b, LEU844g, (VAL726, LEU718, VAL726, ALA743)e
**Luteolin**	−6.1340	2.46	(MET793, MET1002, ASP855, ASN842)a, LEU718g, MET1002g, (LEU844, VAL726, LEU844)e.
**Quercetin**	−6.6886	2.65	(MET793, ASP855)a, THR854d, (LEU718, LEU844)g, (LEU718, VAL726, ALA743, LEU844, VAL726, ALA743, LYS745)e
**Ursolic acid**	−6.2386	2.19	ASN842a, ARG841b, (CYS797, ARG841, CYS797, VAL726)e
**2β-hydroxy-ursolic acid**	−5.5089	1.91	ASP800a, ASP800b, (VAL726, VAL726, ALA743, ALA743, ARG841, LEU844, LEU799, ARG841, LEU718, LEU844, LEU792, MET793, LEU844)e.
**Oleanolic acid**	−6.0885	2.13	(ASN842, LEU718)a, ARG841b, (CYS797, ARG841, CYS797, VAL726)e.
**PubChem ID: 24880017** **(Control)**	−10.1349	3.55	(THR790, MET793)a, (ALA743, LEU788, GLN791)b, (CYS775, ARG776)h, LYS745c, (THR854, ASP855)d, (THR790, LEU844, PHE856)g, (VAL726, MET766, LEU777, LYS745, LEU788,VAL726, ALA743, LYS745, ALA743)e

a = conventional H-bond, b = carbon–H-bond, c = electrostatic (Pi-cation), d = H-bond (Pi-donor/acceptor), e = alkyl/alkyl Pi, g = Pi-sigma, Pi-Pi-stacking, Pi-Pi-T-shape, h = Halogen (Fluorine).

**Table 6:** Docking binding energy scores (in kcal/mol), SILE values, and interacting residues of key *M. officinalis* compounds docked with the transcription factor p65 NF-kB (RELA).

Compound	Docking Score	Sile Value	Interacting Amino Acid Residues
**Isoquercitrin**	−6.778	**2.37**	(LYS37, TYR36, GLY44)a, (ARG41, GLN119, ARG41, GLN119, SER42)b
**Luteolin**	−5.168	2.07	(CYS120, LYS122, ASP185)a, TYR36d, (CYS120, LYS123, LEU154)e
**Quercetin**	−5.322	2.11	GLU89a, ARG124c, GLN119d, (LYS37, LYS122)e
**Ursolic acid**	−4.913	1.72	TYR36a, (LYS123, ALA188, PRO189, LYS122, LYS123)e
**2β-hydroxy-ursolic acid**	−4.514	1.57	LYS123e
**Oleanolic acid**	−4.758	1.67	(ALA188, PRO189, LYS123)e
**PubChem ID: 9881652 (Control)**	−5.715	2.36	(TYR36, LYS122)a, (LYS123, ASP185, LEU154)b, LYS123e

a = conventional H-bond, b = carbon–H-bond, c = electrostatic (Pi-cation), d = H-bond (Pi-donor/acceptor), e = alkyl/alkyl Pi.

**Table 7:** Docking binding energy scores (in kcal/mol), SILE values, and interacting residues of key *M. officinalis* compounds docked with GSK-3B.

Compound	Docking Score	Sile Value	Interacting Amino Acid Residues
**Isoquercitrin**	−6.830	**2.39**	VAL135a, PRO136a, ILE62e
**Luteolin**	−5.900	2.37	(VAL135, ARG141, ASP133)a, LEU188g, (ALA83, VAL110, CYS199)e
**Quercetin**	−5.875	2.32	(VAL135, GLU97)a, (CYS199, ASP200)d, (LEU132, LEU188, MET101)g, (VAL70, CYS199, ALA83, LYS85, VAL110)e
**Ursolic acid**	−6.616	2.32	PRO136a, PRO136b, (VAL70, CYS199, ILE62, ARG141, VAL70, LYS85, PHE67)e
**2β-hydroxy-ursolic acid**	−6.540	2.27	(VAL70, ALA83, LEU188, CYS199, LEU132)e
**Oleanolic acid**	−5.917	2.07	(VAL70, ALA83, LEU188, CYS199, LYS85, LEU132)e
**PubChem ID: 56643097 (Control)**	−8.139	2.61	VAL135a, ASP133b, CYS199d, (LEU188, PHE67)g, (VAL70, LYS85, ILE62, ALA83, VAL110)e

a = conventional H-bond, b = carbon–H-bond, d = H-bond (Pi-donor/acceptor), e = alkyl/alkyl Pi, g = Pi-sigma, Pi-Pi-stacking, Pi-Pi-T-shaped.

**Table 8:** Docking binding energy scores (in kcal/mol), SILE values and interacting residues of key *M. officinalis* compounds docked with protein tyrosine phosphatase 1B (PTP1B).

Compound	Docking Score	Sile Value	Interacting Amino Acid Residues
**Isoquercitrin**	−7.123	**2.50**	ASP48a, ASP48b, LYS120c, TYR46d, (TYR46, PHE182)g, (ALA217, LYS120)e
**Luteolin**	−5.370	2.15	ARG221a, (LYS120, TYR46)d, (PHE182, ALA217, LYS120)g
**Quercetin**	−5.955	2.36	ARG221a, LYS120c, TYR46d, PHE182g, (ALA217, LYS120)e
**Ursolic acid**	−4.246	1.49	(TYR20, TYR46)e
**2β-hydroxy-ursolic acid**	−4.613	1.60	ARG24a, GLY259b, MET258e
**Oleanolic acid**	−4.095	1.43	LYS120b, (TYR46, PHE182)e
**PubChem ID: 91826021** **(Control)**	−9.003	3.43	(ARG221, ARG221, ASP181)c, (PHE182, SER216, ALA217, ILE219, GLY220, ARG221, ARG221, ASP48)a, (ALA217, PHE182)e

a = conventional H-bond, b = carbon–H-bond, c = electrostatic (Pi-cation), d = H-bond (Pi-donor/acceptor), e = alkyl/alkyl Pi, g = Pi-sigma, Pi-Pi-stacking, Pi-Pi-T-shaped.

**Table 9:** Docking binding energy scores (in kcal/mol), SILE values, and interacting residues of key *M. officinalis* compounds docked with the peroxisome proliferator-activated receptor alpha (PPARα).

Compound	Docking Score	Sile Value	Interacting Amino Acid Residues
**Isoquercitrin**	−7.805	**2.73**	CYS275a, (CYS276, THR279, TYR334)b, (VAL332, TYR334)g, (CYS275, CYS276, ILE339, ALA333)e
**Luteolin**	−5.935	2.38	THR279a, ILE272b, (CYS275, CYS276)d, VAL332g, (ILE272, VAL332, ILE241, ALA250, CYS275, VAL332, ALA333)e
**Quercetin**	−6.136	2.43	(CYS276, CYS276, THR279)a, (THR279, TYR334, TYR334)b, (VAL332, TYR334, CYS275)g, (CYS276, CYS275, CYS276, VAL332, ILE339, VAL332)e
**Ursolic acid**	−4.570	1.60	(THR279, CYS275)a, (LEU254, CYS275, CYS275, CYS275, VAL332, ALA333, TYR334, LEU254, LEU254, CYS275, ILE241, VAL332)e
**2β-hydroxy-ursolic acid**	−4.108	1.43	(CYS275, CYS278, VAL332, ALA333, CYS278)e
**Oleanolic acid**	−3.365	1.18	GLU251a, (ALA250, LEU254, CYS275, CYS276, VAL332, ALA333, ILE339, MET330, CYS276, MET330, MET355, CYS276, LEU254)e
**PubChem ID: 60151560 (Control)**	−8.173	2.92	(SER280, TYR314, TYR464)a, SER280b, CYS276d, (VAL332, MET355)g, (CYS276, MET330, LEU344, MET355, VAL444, CYS278, PHE273, TYR334, HIS440, VAL332, ILE339, CYS275, ALA333)e

a = conventional H-bond, b = carbon–H-bond, d = H-bond (Pi-donor/acceptor), e = alkyl/alkyl Pi, g = Pi-sigma, Pi-Pi-stacking, Pi-Pi-T-shaped.

**Table 10:** Molecular interactions between isoquercitrin and AKT1, PIK3R1, and INSR.

Target Protein	Interacting Residues	Distance (Å)	Category	Type
ATK1 (PDBID:3O69)	TYR272	2.03	Hydrogen bond	Conventional hydrogen bond
	GLN79	2.35	Hydrogen bond	Conventional hydrogen bond
	SER205	2.05	Hydrogen bond	Conventional hydrogen bond
	THR211	2.94	Hydrogen bond	Conventional hydrogen bond
	ILE290	2.20	Hydrogen bond	Conventional hydrogen bond
	GLY294	3.77	Hydrogen bond	Carbon hydrogen bond
	TYR272	2.85	Hydrogen bond	Carbon hydrogen bond
	TYR272	2.86	Hydrogen bond	Carbon hydrogen bond
	THR82	2.67	Hydrogen bond	Carbon hydrogen bond
	TRP80	3.94	Hydrophobic	Pi-Pi stacked
	TRP80	4.28	Hydrophobic	Pi-Pi stacked
	TRP80	4.81	Hydrophobic	Pi-Pi stacked
	TRP80	3.97	Hydrophobic	Pi-Pi stacked
	LEU264	5.41	Hydrophobic	Pi-Alkyl
	VAL270	4.68	Hydrophobic	Pi-Alkyl
	LYS268	4.40	Hydrophobic	Pi-Alkyl
	VAL270	4.97	Hydrophobic	Pi-Alkyl
	LEU210	4.73	Hydrophobic	Pi-Alkyl
	LEU264	5.03	Hydrophobic	Pi-Alkyl
PIK3R1 (PDBID:2IUG)	ARG19	3.01	Hydrogen bond	Conventional hydrogen bond
	ARG19	3.11	Hydrogen bond	Conventional hydrogen bond
	THR48	2.54	Hydrogen bond	Conventional hydrogen bond
	ASP16	2.20	Hydrogen bond	Conventional hydrogen bond
	ASP16	1.72	Hydrogen bond	Conventional hydrogen bond
	SER18	3.44	Hydrogen bond	Carbon hydrogen bond
	SER40	2.48	Hydrogen bond	Carbon hydrogen bond
	SER40	2.67	Hydrogen bond	Carbon hydrogen bond
	THR48	3.02	Hydrogen bond	Carbon hydrogen bond
	THR48	3.08	Hydrogen bond	Carbon hydrogen bond
	GLU20	3.81	Electrostatic	Pi-Anion
	SER18	3.82	Hydrophobic	Pi-Sigma
	ARG19	4.50	Hydrophobic	Pi-Alkyl
INSR (PDBID:6PXW)	SER85	2.99	Hydrogen bond	Conventional hydrogen bond
	ARG114	3.02	Hydrogen bond	Conventional hydrogen bond
	GLN249	2.99	Hydrogen bond	Conventional hydrogen bond
	ARG331	2.73	Hydrogen bond	Conventional hydrogen bond
	ARG331	2.97	Hydrogen bond	Conventional hydrogen bond
	ASP250	1.94	Hydrogen bond	Conventional hydrogen bond
	ASP250	1.93	Hydrogen bond	Conventional hydrogen bond
	GLN328	2.53	Hydrogen bond	Conventional hydrogen bond
	CYS284	1.93	Hydrogen bond	Conventional hydrogen bond
	CYS284	2.24	Hydrogen bond	Conventional hydrogen bond
	SER85	2.86	Hydrogen bond	Carbon hydrogen bond
	ASP250	2.64	Hydrogen bond	Carbon hydrogen bond
	ARG86	3.50	Electrostatic	Pi-Cation
	ARG86	3.84	Electrostatic	Pi-Cation
	PRO286	5.17	Hydrophobic	Pi-Alkyl

**Table 11:** Molecular interactions between isoquercitrin and EGFR, NF-κB, GSK3B, PTP1B, and PPARα.

Target Protein	Interacting Residues	Distance (Å)	Category	Type
**EGFR** (PDBID:2RGP)	LYS745	3.31	Hydrogen bond	Conventional hydrogen bond
	MET793	3.07	Hydrogen bond	Conventional hydrogen bond
	ASN842	2.73	Hydrogen bond	Conventional hydrogen bond
	ASP855	2.48	Hydrogen bond	Conventional hydrogen bond
	MET793	2.31	Hydrogen bond	Conventional hydrogen bond
	THR854	2.81	Hydrogen bond	Carbon hydrogen bond
	LEU844	3.73	Hydrophobic	Pi-Sigma
	LEU844	4.00	Hydrophobic	Pi-Sigma
	VAL726	4.73	Hydrophobic	Pi-Alkyl
	LEU718	5.23	Hydrophobic	Pi-Alkyl
	VAL726	5.23	Hydrophobic	Pi-Alkyl
	ALA743	4.53	Hydrophobic	Pi-Alkyl
**NF-κB p65** (PDBID:1NFI)	LYS37	2.23	Hydrogen bond	Conventional hydrogen bond
	TYR36	2.17	Hydrogen bond	Conventional hydrogen bond
	GLY44	2.71	Hydrogen bond	Conventional hydrogen bond
	GLY44	2.17	Hydrogen bond	Conventional hydrogen bond
	ARG41	2.31	Hydrogen bond	Carbon hydrogen bond
	GLN119	2.38	Hydrogen bond	Carbon hydrogen bond
	ARG41	2.60	Hydrogen bond	Carbon hydrogen bond
	GLN119	2.67	Hydrogen bond	Carbon hydrogen bond
	SER42	2.83	Hydrogen bond	Carbon hydrogen bond
**GSK3B** (PDBID:4AFJ)	VAL135	2.70	Hydrogen bond	Conventional hydrogen bond
	PRO136	1.77	Hydrogen bond	Conventional hydrogen bond
	ILE62	4.76	Hydrophobic	Pi-Alkyl
**PTP1B** (PDBID:4Y14I)	ASP48	3.03	Hydrogen bond	Conventional hydrogen bond
	ASP48	2.62	Hydrogen bond	Carbon hydrogen bond
	ASP48	2.31	Hydrogen bond	Carbon hydrogen bond
	LYS120	3.67	Electrostatic	Pi-cation
	TYR46	4.03	Hydrogen bond	Pi-donor hydrogen bond
	TYR46	4.78	Hydrophobic	Pi-Pi stacked
	PHE182	4.41	Hydrophobic	Pi-Pi stacked
	PHE182	4.00	Hydrophobic	Pi-Pi stacked
	ALA217	4.58	Hydrophobic	Pi-alkyl
	ALA217	4.14	Hydrophobic	Pi-alkyl
	LYS120	5.44	Hydrophobic	Pi-alkyl
**PPARα** (PDBID:6LXC)	CYS275	2.57	Hydrogen bond	Conventional hydrogen bond
	CYS276	3.48	Hydrogen bond; other	Pi-donor hydrogen bond; Pi-Sulfur
	THR279	2.98	Hydrogen bond	Pi-donor hydrogen bond
	TYR334	3.97	Hydrogen bond	Pi-donor hydrogen bond
	VAL332	3.68	Hydrophobic	Pi-Sigma
	VAL332	3.92	Hydrophobic	Pi-Sigma
	TYR334	4.93	Hydrophobic	Pi-Pi T-shaped
	CYS275	4.81	Hydrophobic	Pi-Alkyl
	CYS276	5.12	Hydrophobic	Pi-Alkyl
	ILE339	5.13	Hydrophobic	Pi-Alkyl
	VAL332	5.04	Hydrophobic	Pi-Alkyl
	ALA333	4.87	Hydrophobic	Pi-Alkyl

## Data Availability

The raw data supporting the conclusions of this article will be made available by the authors upon request.

## Supplementary Materials

The following supporting information can be downloaded at https://www.mdpi.com/article/10.3390/plants14182828/s1, Table S1: ADME/Drug-likeness predictions of M. officinalis phytochemicals (compounds selected for the network pharmacology study are in bold); Table S2: List of biological targets predicted to be associated with M. officinalis selected compounds; Table S3: List of biological targets predicted to be associated with both M. officinalis compounds and T2DM; Table S4: List of predicted T2DM targets associated with M. officinalis compounds, used to construt the compound-target (CT) network; Table S5: Topological parameters of the predicted common targets in the protein-protein interaction network (showing result of targets with Degree ≥ 4( the median degree of the network)); Table S6: Ranking of predicted targets enriched in the Target-Pathway networks by topological parameter, degree; Table S7: Validation of the the London dG / GBVI/WSA dG docking scoring functions In MOE using target structures wth co-crystalized ligands; Table S8: RMSD values obtained in MD simulations investigating isoquercitrin in complex with AKT1, EGFR, INSR, PIK3R1, PPARA, GSK3B, NFKB, PTP1B; Table S9: RMSF values obtained in MD simulations investigating isoquercitrin in complex with AKT1, EGFR, INSR, PIK3R1, PPARA, GSK3B, NFKB, PTP1B; Table S10: Rg values obtained in MD simulations investigating isoquercitrin in complex with AKT1, EGFR, INSR, PIK3R1, PPARA, GSK3B, NFKB, PTP1B; Table S11: SASA values obtained in MD simulations investigating isoquercitrin in complex with AKT1, EGFR, INSR, PIK3R1, PPARA, GSK3B, NFKB, PTP1B; Figure S1: Insulin signaling pathway in muscle/liver cell, showing the enriched targets in red (data on KEGG graph, rendered by Pathview); Figure S2: Obesity and insulin resistance in muscle cell, showing the enriched targets in red (data on KEGG graph, rendered by Pathview); Figure S3: Obesity and insulin resistance in liver cell, showing the enriched targets in red (data on KEGG graph, rendered by Pathview).
