# Supplementary material for: Network Pharmacology, Molecular Docking and Molecular Dynamics Studies to Predict the Molecular Targets and Mechanisms of Action of Melissa officinalis Phytoconstituents in Type-2 Diabetes Mellitus
Source: Plants (Basel). 2025 Sep 10;14(18):2828. doi: 10.3390/plants14182828 (PMC12473937; doi:10.3390/plants14182828)
Supplement: Supplementary file 1 [file plants-14-02828-s001.zip › 0424_SM_figures S1-S3.docx]

Supplementary Material

Network Pharmacology, Molecular Docking and Molecular Dynamics Studies to Predict the Molecular Targets and Mechanisms of Action of *Melissa officinalis* phytoconstituents in Type-2 Diabetes Mellitus

Chimaobi J. Ononamadu ^1,2^ , Ziyad Ben Ahmed^3,4^, and Veronique Seidel ^1,^*

^1^ Natural Products Research Laboratory, Strathclyde Institute of Pharmacy and Biomedical Sciences, University of Strathclyde, Glasgow, UK.

^2^ Natural Product Research Group, Department of Biochemistry and Forensic Science, Nigeria Police Academy, Wudil, Kano State, Nigeria.

^3^ Laboratoire des Sciences Fondamentales, Université Amar Telidji, Laghouat, Algeria

^4^ Department of Analytical Chemistry, Applied Chemometrics and Molecular Modelling, Vrije Universiteit Brussel (VUB), Brussels, Belgium

***** Correspondence: Veronique Seidel ([veronique.seidel@strath.ac.uk](mailto:veronique.seidel@strath.ac.uk))


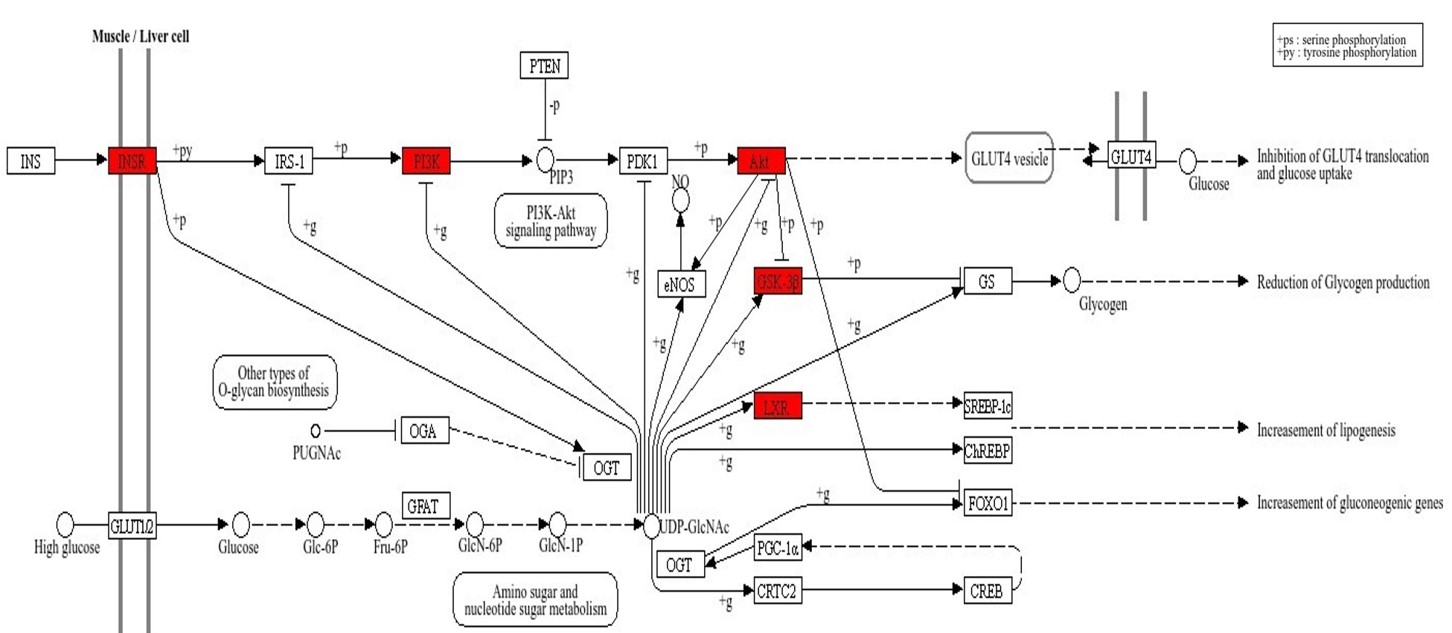


**Figure S1:** Insulin signaling pathway in muscle/liver cell, showing the enriched targets in red (data on KEGG graph, rendered by Pathview).

**
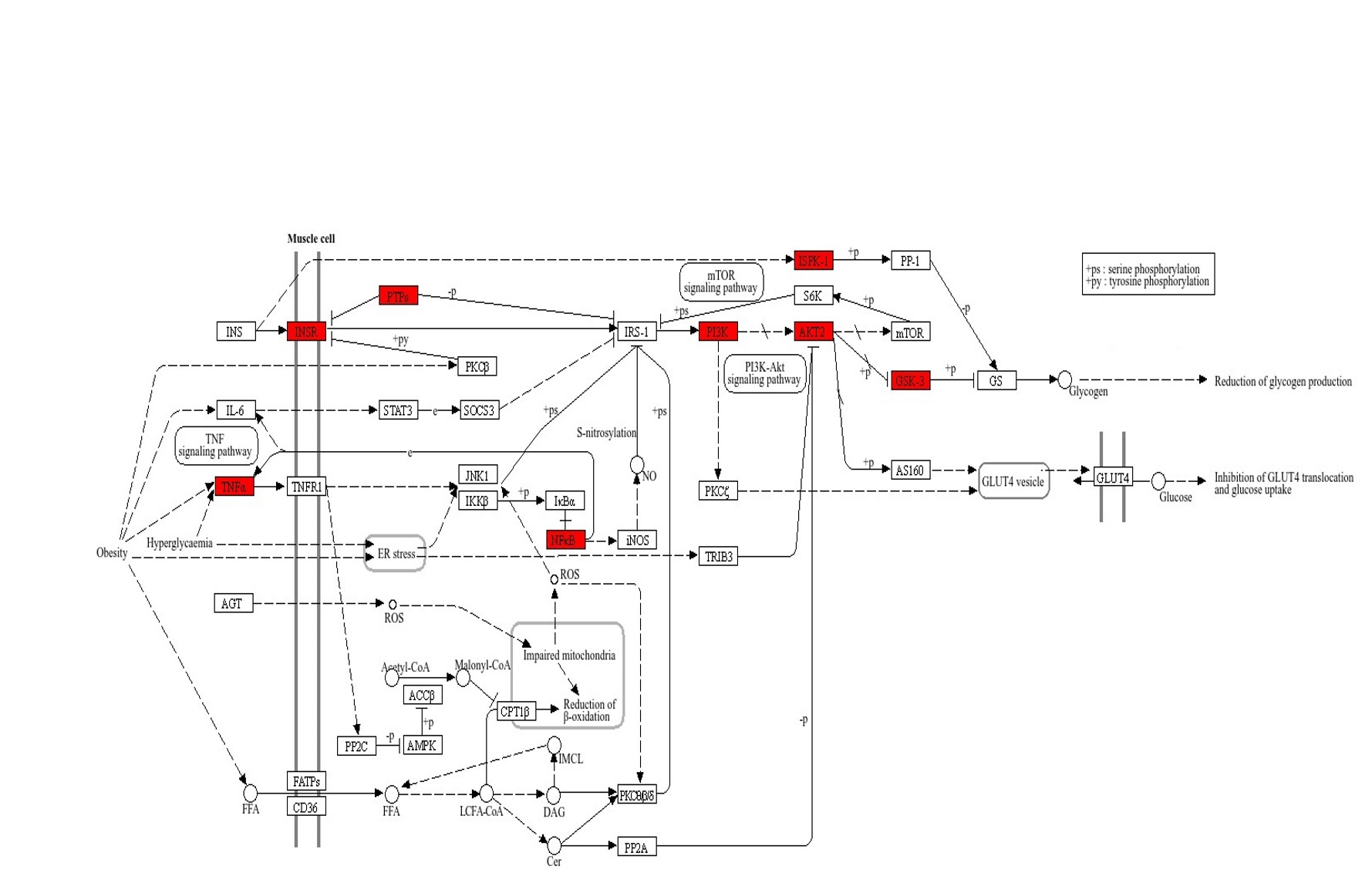
Figure S2**: Obesity and insulin resistance in muscle cell, showing the enriched targets in red (data on KEGG graph, rendered by Pathview).

**
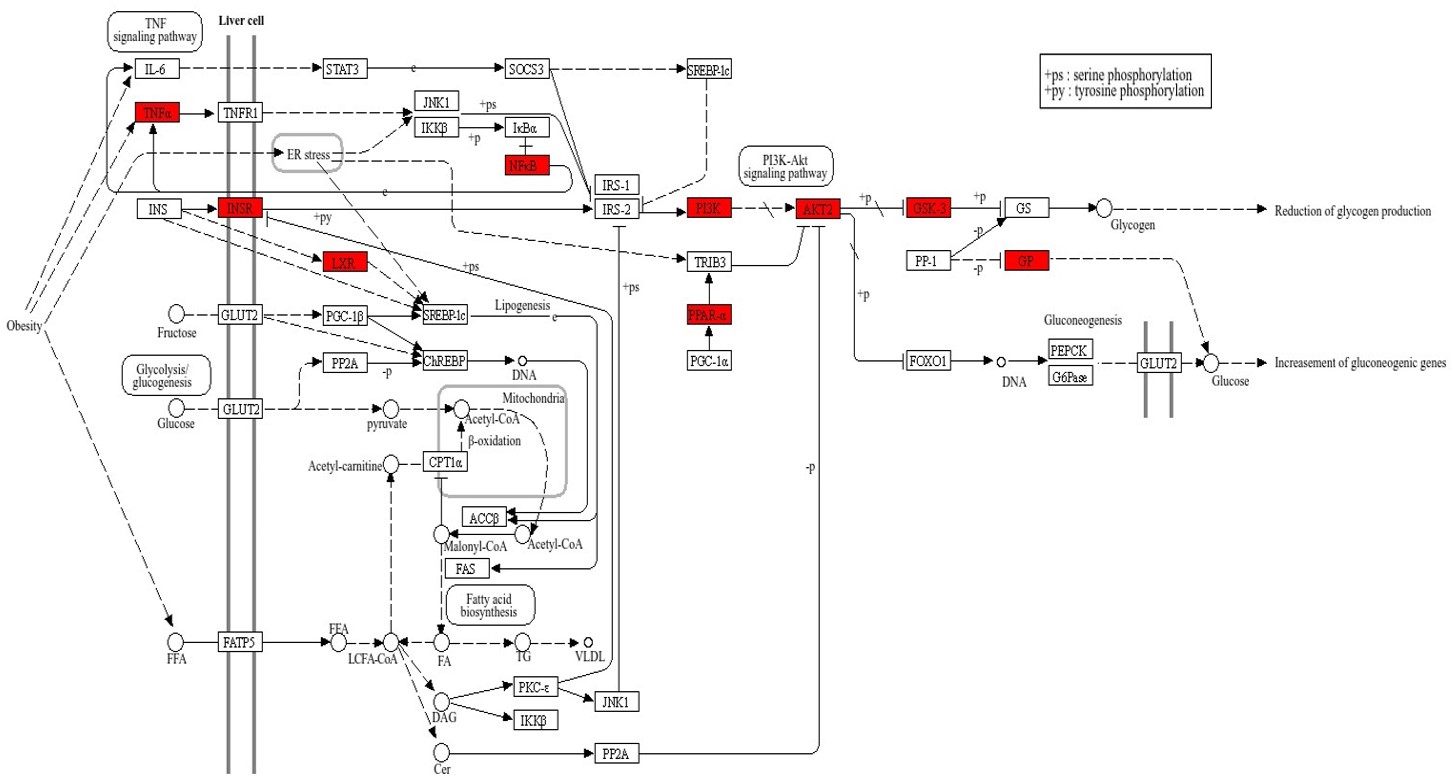
Figure S3**: Obesity and insulin resistance in liver cell, showing the enriched targets in red (data on KEGG graph, rendered by Pathview).
